# Ball and socket ankle joint in connection with bilateral tarsal synostosis in a boy with congenital absence of the portal vain: a novel malformation complex

**DOI:** 10.1186/1757-1626-1-76

**Published:** 2008-08-08

**Authors:** Shahin Zandieh, Anosheh Vakli-Adli, Josef Hochreiter, Franz Grill, Klaus Klaushofer, Ali Al Kaissi

**Affiliations:** 1 Department of Radiology, Hanusch Hospital, Vienna, Austria; 2 Department of Orthopaedic Surgery, St. Vincent's Hospital, Linz, Austria; 3 Ludwig Boltzmann Institute of Osteology, at the Hanusch Hospital of WGKK and, AUVA Trauma Centre Meidling, 4th Medical Department, Hanusch Hospital, Vienna, Austria; 4 Orthopaedic hospital of Speising, Paediatric department, Vienna, Austria

## Abstract

**Background:**

Contracted valgus flat foot in the adolescent is frequently caused by tarsal synostosis or synchondrosis. These synostoses are prevalently symptomatic during adolescence, when by ossifying they block the subtalar joint in valgus. Careful and detailed examinations might reveal additional abnormalities.

**Case presentation:**

A 16-year-old boy of Austrian origin presented with contracted valgus foot associated with tarsal hypomobility and pain. Talonavicular synostosis with ball and socket ankle joint was detected via lateral radiographs and 3 DCT scan. Preoperative laboratory investigations revealed leucocytopenia, and thrombopenia. Computerised abdominal tomography showed portal vein atresia and portopulmonary hypertension.

**Conclusion:**

Clinical research is the corner stone to elucidate the aetiological understandings in patients with malformation complex. The latter is a critical task for the development of scientific bases for preventive strategies. Careful examination for these abnormalities should lead the clinician to earlier referral of patients for additional examination by a specialised medical team. This often enables more focused care for the individual and better characterisation/documentation of the malformation complex. The association of tarsal synostosis and the previously unreported associated occurrences of congenital absence of the portal vein, portopulmonary hypertension, cardiomegaly and splenomegaly have been encountered. We stress that our present patient illustrates and supports the pathophysiological hypotheses that have previously proposed for the concurrent existence of absent portal vein, hepatic nodular hyperplasia and portopulmonary hypertension. Nevertheless, no previous single report signifies the existence of tarsal synostosis in connection with the above-mentioned abnormalities.

## Background

Tarsal coalition represents abnormal fusion between two or more tarsal bones and is a frequent cause of foot and ankle pain. The clinical and radiological features depend on the anatomic location of the coalition. Calcaneonavicular and talonavicular coalitions are the most frequent features, which may result in peroneal spastic flat foot; however they are symptomless in most cases. Talonavicular synostosis in the growing foot results in marked impairment of subtalar range of motion. The development of a ball and socket ankle joint may be caused thereby, if tarsal hypomobility becomes effective at an early stage of growth [1-4]. Tarsal synostosis might occur either as part of a syndromic malformation complex or as an isolated deformity [5-7].

A congenital absence of the portal vein (CAPV) is a rare malformation known as "Abernethy malformation". It is a rare congenital portosystemic shunt in which the blood directly drains into the systemic vein bypassing the liver either through a complete (type 1) or a partial shunt (type 2) [8]. Abnormalities of the portal venous system arise from alterations in the configuration forming the portal vein and also are secondarily associated with a variety of other malformations. Cardiac defects and tumour of the liver have been described as the main presenting features particularly in type I [9,10]. Abernethy malformation has been encountered in a patient with Goldenhar syndrome [11].

## Case presentation

A 16-year-old-boy was referred to the orthopaedic department because of foot and ankle pain associated with tarsal hypomobility. He was the first child of a 29-year-old mother and a non-consanguineous 35-year old father. The pregnancy was uneventful, and the parents knew non-teratogenic exposures. He was born full term, his weight, length and head circumference were around the 50^th ^Percentile. There was no family history of inheritable disorders. His developmental history was within normal limits. His medical history was unremarkable.

**Figure 1:**
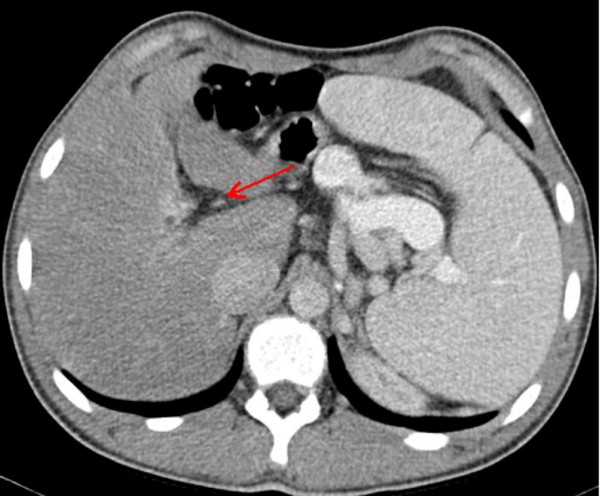
Computerised abdominal tomography showed splenomegaly and absence of the intrahepatic portion of the portal vein (arrow).

**Figure 2:**
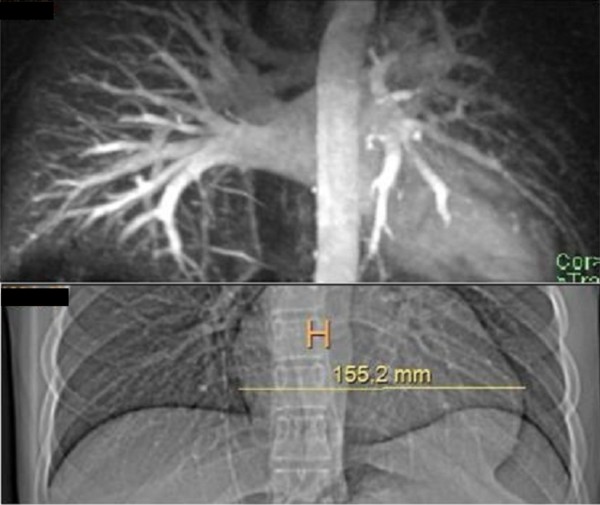
**Computed tomography (CT) of the thorax revealed prominent cardiomegaly and pulmonary vessels**.

**Figure 3:**
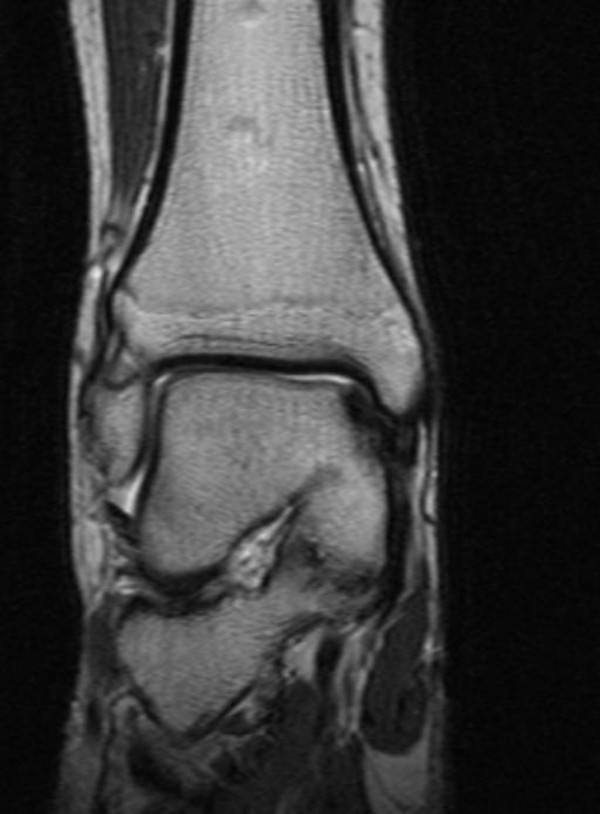
3D coronal CT of the ankle and hid foot showed significant talocalcaneal coalition.

Clinical examination showed a boy with normal height. He did not have dysmorphic facial/skeletal features. He had a normal upper/lower body segments. Examination of the chest and the spine was normal. Hearing and vision were normal. No neurological deficits were elicited. Recently he began to walk with slight supination of the forefeet and complete lack of subtalar movement accompanied with pain. Peroneal spasm, pes planus and calcaneus valgus were notable. Preoperative laboratory investigations revealed leucocytopenia, and thrombopenia. Although, his blood ammonia level was normal and there was no sign of hepatic encephalopathy. Abdominal ultrasonography showed portal hypertension associated with atypical liver haemangioma/adenoma in the right lobe of 2 cm in diameter. Computerised abdominal tomography showed splenomegaly and absence of the intrahepatic portion of the portal vein and a partial portosystemic shunt (figure 1). Computed tomography (CT) of the thorax revealed prominent cardiomegaly and pulmonary vessels in connection with portopulmonary hypertension (fig 2). 3D coronal CT of the ankle and hind foot showed significant talocalcaneal coalition (figure 3). At this stage his parents rejected further investigations such as liver biopsy to further delineate the type of the tumour. We put our patient under conservative treatment.

## Discussion

Congenital tarsal coalition is a diagnosis that is often overlooked in young patients who first present with foot and ankle pain. Most patients present with hindfoot or tarsal pain or stiffness, which is often first noted after weight gain, or increase in athletic activity. A history of repeated ankle sprains or subtalar pain may ignite the symptoms. Tarsal coalition is a common cause of a peroneal spastic flatfoot or rigid flatfoot. The condition does not represent true spasticity, but rather peroneal spasm or adaptive peroneal shortening to adjust for the heel valgus and to maintain the subtalar joint in the least painful position. Congenital tarsal coalition likely results from abnormal differentiation and segmentation of primitive mesenchyme with resultant lack of joint formation [1-5]. Calcaneonavicular and talocalcaneal coalitions are encountered most frequently; fusion at other sites is much less common. There have been a remarkable number of reports describe talonavicular synostosis. Anderson [12] reported a case of talo-navicular fusion in 1880. Lamb [13] described five cases of talo-calcaneo-navicular synostosis in which he observed an interesting abnormality of the ankle joint which had a " ball and socket" appearance and which allowed a few degrees of inversion and eversion of the heel to compensate for the fusion of the subtalar joint. Tarsal fusions were described in connection with syndromic malformation complexes such as spondylocarpotarsal synostosis and with symphalangism [6,7].

John Abernethy [8] based on the postmortem examination of a 10-month-old female who died of unknown causes. Described a congenital diversion of portal blood away from the liver, by either end-to-side or side-to-side shunt.

The diagnosis is most frequently established primarily with ultrasound. CT and MRI are used for further classification of the shunt and assessment of accompanying liver tumors and malformations. In type I shunt, there is complete diversion of the portal blood into the vena cava, with a congenital absence of the portal vein. In type II, the portal vein is intact, but the portal blood is diverted into the vena cava through a side-to-side, extrahepatic communication. In addition to this classification, there is a clear clinical distinction between patients with type I and type II shunts based on the analysis of the reported cases. Type I shunt, is almost congenital, such patients are young girls at the time of presentation. Despite the presence of high ammonemia, encephalopathy is usually not so marked in these patients. Type II shunt is acquired rather than congenital, patients are usually presented with the disorder at a middle or late-middle age. Encephalopathy with high serum ammonia level of unknown cause and a vascular anomaly discovered via vigorous investigations do occur [9].

Congenital absence of the portal vein (CAPV) has been reported to occur in connection with multiple congenital anomalies such as malrotation, duodenal atresia, biliary artesia, and annular pancreas for the gastrointestinal tract and situs inversus, atrial septal defect (ASD), ventricular septal defects (VSD), and patent ductus arteriosus (PAD) for cardiovascular systems [10]. Congenital absence of the portal vein has been encountered in a patient with oculoauriculovertebral dysplasia (Goldenhar syndrome) [11]. For the association between absent portal vein and pulmonary hypertension, it has been suggested that vasoactive substances bypassing the liver through portosystemic collaterals can cause pulmonary arterial spasm and thrombosis, which is a condition called portopulmonary hypertension [14].

We conclude that, as far as can be determined this is the first clinical report describing a combination of ball and socket ankle joint in a patient with Abernethy malformation. The coincidence of tarsal synostosis and congenital absence of the portal vein might be caused by a single gene, but this might be an occasional mutation because of isolated occurrence of our case.

## Abbreviations

CAPV: Congenital absence of the portal vein; ASD: Atrial septal defect; VSD: Ventricular septal defects; PAD: Patent ductus arteriosus; CT: Computerised tomography.

## Competing interests

The authors declare that they have no competing interests.

## Authors' contributions

All of the authors were involved in the clinico-radiographic assessment and finalising the paper. All authors have red and approved the final version of the paper.

## Consent

Written informed consent was obtained from the parents for the purpose of publication of the manuscript and figures of their child. A copy of the written consent is available for review by the editor-in-Chief of this journal.
